# Association of maternal omega-6 fatty acid intake with infant birth outcomes: Korean Mothers and Children’s Environmental Health (MOCEH)

**DOI:** 10.1186/s12937-018-0353-y

**Published:** 2018-04-21

**Authors:** Eunjung Lee, Hyesook Kim, Hyejin Kim, Eun-Hee Ha, Namsoo Chang

**Affiliations:** 1 Department of Nutritional Science and Food Management, Ewha Womans University, Seoul, South Korea; 2 Department of Nutrition Consultation, Seoul National University Hospital, Healthcare System Gangnam Center, Seoul, South Korea; 3 Department of Occupational and Environmental Medicine, Ewha Womans University, College of Medicine, Seoul, South Korea

**Keywords:** Pregnancy outcome, Omega-6 fatty acids, Pregnant women, Birth weight

## Abstract

**Background:**

Maternal fatty acids (FAs) intake has an effect on birth weight, birth length, and gestational age, as fetal development is entirely dependent on the maternal essential FA supply. This study aimed to identify the association between the maternal intake of FAs and birth outcomes among pregnant women who participated in the Mothers and Children’s Environmental Health (MOCEH) prospective cohort study in South Korea.

**Methods:**

A total of 1407 pregnant women, aged 30.2 ± 3.7 years, at 12 to 28 weeks’ gestation were recruited between August 2006 and December 2010. Their dietary intake during pregnancy was investigated by the 1-day 24-h dietary recall method. The pregnancy outcome data—namely infant’s gestational age, birth weight, and birth length—were analyzed for their associations with their mothers’ intake of FAs.

**Results:**

When adjusted for confounding factors, multiple regression analysis revealed adverse effects on birth weight (*P* = 0.031) and birth length (*P* = 0.025) with high maternal intake of omega-6 FAs. In the multiple logistic regression analysis, the odds ratio (OR) for the risk of being below the 10th percentile for birth weight was higher in the highest quintile (Q5) compared to the lowest quintile (Q1) of omega-6 FA intake levels (OR = 2.444; 95% CI = 1.038–5.751; P for trend = 0.010). Also, the OR for being above the 90th percentile of birth length was lower in the highest quintile (Q5) compared to that in the lowest quintile (Q1) of omega-6 FA intake (OR = 0.432; 95% CI = 0.211–0.884; P for trend = 0.020). However, the maternal intake of omega-3 FAs was not related to gestational age, birth weight, or birth length.

**Conclusions:**

A high maternal omega-6 FA intake was negatively associated with birth weight and birth length.

## Background

Many studies have demonstrated a link between maternal fatty acids (FAs) intake and pregnancy outcomes, including infant’s birth weight and birth length. Despite these findings, the mechanisms underlying the correlation between maternal FAs intake and fetal size have not been fully elucidated. Nevertheless, deficiencies in the levels of the mothers’ FAs intake could have negative outcomes on the birth weight, birth length, and gestational age of the infant, as fetal development is entirely dependent on the maternal supply of essential FAs [1]. Long-chain polyunsaturated fatty acids (LCPUFAs), such as docosahexaenoic acid (DHA; 22:6 *n*-3) and arachidonic acid (ARA; 20:4 *n*-6), are important for the development of the fetus during pregnancy [2–4].

Omega-3 FAs have received considerable attention in exploring the correlations of maternal FAs intake with birth weight. Several studies have indicated that gestational age, birth weight, and birth length increase when the consumption of omega-3 FAs during pregnancy increases [5–11]. However, other studies have shown conflicting results [12–14].

Nonetheless, few investigations have focused on the relationship between maternal omega-6 FAs intake and birth outcomes, with only one literature report, to date, which pertained to pregnant women in India. It found that the birth weight was low at both low and high intakes of maternal linoleic acid (LA; 18:2 *n*-6), suggesting an inverted U–shaped relationship [15]. In addition, infants born to the group whose maternal diet had a higher omega-6/omega-3 ratio had lower birth weights, and vice versa. Other researchers have noted that higher levels of omega-6 FAs and ARA in maternal blood significantly lowered birth weight in early pregnancy and that elevated levels of ARA in late pregnancy were also associated with decreasing birth weight [16, 17]. Furthermore, Meher and coworkers observed that total omega-6 FA and ARA levels in maternal erythrocytes were higher in mothers delivering low-birth-weight babies (< 2.5 kg) than those whose newborns weighed more than 2.5 kg [18].

The preceding examples suggest that there is a high correlation between maternal FAs intake and fetal weight but, except for the assessment of pregnant women for FAs intake, there have been no in-depth studies of Korean women in this regard [19]. Also, the only study that measured total omega-3 and omega-6 FAs intake among pregnant women in Korea was limited, owing to the lack of an FA database for estimation of FA intake levels in the Korean population, at that time.

Therefore, the purpose of this study was to estimate the FAs intake of pregnant Korean women and to investigate the relationship between FAs intake and pregnancy outcomes, using a prospective sample from the Mothers and Children’s Environmental Health (MOCEH) study [20].

## Methods

### Study design and participants

Study subjects were 1751 midstage (12–28 weeks of gestation) pregnant women enrolled in the MOCEH study [20] between August 2006 and December 2010. Of these 1751 women, 69 were excluded from our study (31 who were carrying twins, 35 who had had a miscarriage and/or were carrying a fetus with a congenital anomaly, and 3 who had an intrauterine growth restriction). Of the remaining 1682 women, 39 more were excluded (38 who had complications such as hypertension or/and diabetes and 1 had dropped out and whose pregnancy outcome data were not available). Among the remaining 1643 women, 236 more were excluded (229 whose dietary intake data were not collected and 7 whose total energy consumption was less than 500 or more than 4000 kcal per day). Therefore a total of 1407 women were finally included in the analysis. The study protocols and consent forms were approved by the three institutional review boards at the Ewha Womans University School of Medicine, the Dankook University Hospital, and the Ulsan University Hospital. All subjects provided informed consent to participate in the study, and they were questioned by trained interviewers.

General information on demographic factors, socioeconomic factors, and health-related behaviors was collected at a regular midtrimester checkup through an individual interview. Detailed data included subject’s age, height, weight, parity, education level, family monthly income, dietary supplement use, smoking behavior, and alcohol. Pre-pregnancy body mass index (BMI) was calculated as weight (kg) divided by height (m^2^) by employing self-reported height and weight values before conception. Education levels were categorized as less than high school graduation, university level graduation, or higher than university level graduation. Family monthly income was categorized as less than US$2000, US$2000 to US$4000, and greater than US$4000. Cigarette smoking was classified as current smoker or nonsmoker, and alcohol drinking was categorized into current drinker or nondrinker. Exposure to secondhand smoke was defined as living with someone who smoked or working in an environment where smoking was permitted. Smoking and drinking were assessed by self-report.

### Dietary assessment

Dietary intake data at midpregnancy were collected by 24-h recall for intake the day before the participant’s visit to the center. For the 24-h recall method, experienced, well-trained dietary interviewers asked the respondents to recall and describe all the foods and beverages they had consumed over the preceding 24 h. The food items most frequently mentioned were pictured on a chart which was shown to the participants to help them report more accurately. These nutrients in these dietary intakes were assessed using a computerized nutrient intake assessment software program (CAN-Pro 4.0, Korean Nutrition Society, Seoul, Korea) [21]. For the assessment of maternal FA intake, the CAN-Pro 4.0 FA database was supplemented by the step of database development [22], which covered about 93% of total FA intake of the 24-h recall method of the subjects.

### Infant birth outcome

Information on neonates such as gestational age, birth weight, birth length, and gender was obtained from birth records. Gestational age at delivery was counted from the last menstrual period and examined by ultrasound.

### Statistical analysis

General characteristics of the pregnant women and their neonates were expressed as means with standard deviations or as numbers with percentages. Multiple linear regression analysis was performed to evaluate the association between maternal FA intake and pregnancy outcome, such as birth weight and birth length. Multiple logistic regression analysis was also performed to estimate the odds ratios (ORs) and 95% confidence intervals (CIs) for the risk of less than 10th percentile or more than 90th percentile for birth weight and length across dietary FA intake level (quintiles). The covariates included maternal age [23], pre-pregnancy BMI [24–28], level of education, neonate’s gender [29], log-transformed maternal urinary cotinine level (ln) [30], gestational age at delivery [31, 32], and energy intake. In analyzing the association between maternal FA intake and birth length, vitamin C intake was included among the covariates because it has been reported that maternal vitamin C intake is associated with birth length [33]. All the statistical analyses were conducted using SAS 9.4 software (SAS Institute Inc., Cary, NC, USA); the level of significance was set at *P* < 0.05.

## Results

### General characteristics

The mean (SD) maternal age at study enrollment was 30.2 ± 3.7 years, and prepregnancy BMI was 21.3 ± 3.1 kg/m^2^ (Table 1). Approximately 56.9% of subjects took nutritional supplements. The average energy and fat intakes of the women were respectively 1760.4 ± 494.9 kcal and 46.2 ± 23.7 g per day.

**Table 1:** General characteristics of study subjects

	*n*	Mean ± SD		Range
Pregnant women				
Age, y	1326	30.2 ± 3.7		18–45
Height, cm	1300	161.2 ± 4.8		146–180
Weight, kg				
Pre-pregnancy	1316	55.3 ± 8.5		39–94
Pregnancy	1258	59.6 ± 9.3		37–117
Body mass index, kg/m^2^				
Pre-pregnancy	1292	21.3 ± 3.1		15.8–42.8
Pregnancy	1248	22.9 ± 3.4		15.8–43.5
Parity	1138			
Primigravida, *n*(%)			638(56.1)	
Multigravida, *n*(%)			500(43.9)	
Education, *n*(%)	1276			
≤ High school			342(26.8)	
≤ University			852(66.8)	
≥ Graduate school			82(6.4)	
Family monthly income(USD), *n*(%)	1253			
< 2000			339(27.1)	
2000 ≤ and < 4000			675(53.9)	
≥ 4000			239(19.1)	
Supplement user, *n*(%)	1405		799(56.9)	
Cigarette smoker, current, *n*(%)	1315		12(0.9)	
Secondhand smoking exposer, *n*(%)	1237		179(14.5)	
Urinary cotinine, μg/g creatinine	1323	45.3 ± 312.1		0–5882
Alcohol drinker, current, *n*(%)	1237		67(5.4)	
Daily dietary intake	1407			
Energy, kcal/day		1760.4 ± 494.9		
Fat, g/day		46.2 ± 23.7		
Neonates				
Gestational age at delivery, days	1262	274.8 ± 10.3		191–294
Birth weight, g	1291	3262.5 ± 427.0		500–4650
Birth length, cm	1201	50.5 ± 2.5		26–38.5
Gender	1296			
Boys, *n*(%)			674(52.0)	
Girls, *n*(%)			622(48.0)	

### Fatty acid intake of subjects

The average total FA intake of subjects was 43.5 ± 25.5 g per day. Saturated, monounsaturated, and polyunsaturated FA intakes were respectively 14.8 ± 10.0 g, 16.6 ± 11.2 g, and 11.9 ± 6.7 g per day. Total omega-3 FA intake was 1.47 ± 1.45 g per day, and the intake of DHA was 0.30 ± 0.83 g per day. Total omega-6 FA intake was 10.73 ± 6.34 g per day. The ratio of omega-6 to omega-3 FAs was 9.82 ± 7.00 (Table 2).

**Table 2:** Daily maternal fatty acids intake (*n* 1407)

	Mean ± SD*	Range*
TFAs, g	43.51 ± 25.53	0.48–205.36
SFAs, g	14.78 ± 9.99	0.07–85.37
MUFAs, g	16.60 ± 11.15	0.12–90.11
PUFAs, g	11.90 ± 6.70	0.29–54.82
Total n-3 FAs, g	1.47 ± 1.45	0.04–11.88
18:3 *n*-3, g	1.05 ± 0.78	0.02–8.06
20:4 *n*-3, g	0.00 ± 0.02	0.00–0.00
20:5 *n*-3, g	0.14 ± 0.35	0.00–3.43
22:5 *n*-3, g	0.01 ± 0.03	0.00–1.00
22:6 *n*-3, g	0.30 ± 0.83	0.00–9.76
Total *n*-6 FAs, g	10.73 ± 6.34	0.44–50.88
18:2 *n*-6, g	9.99 ± 6.09	0.03–49.79
20:2 *n*-6, g	0.07 ± 0.12	0.00–1.00
20:3 *n*-6, g	0.09 ± 0.08	0.00–1.00
20:4 *n*-6, g	0.06 ± 0.10	0.00–0.85
22:5 *n*-6, g	0.02 ± 0.10	0.00–1.00
*n*-6/*n*-3	9.82 ± 7.00	0.31–63.00

TFAs, Total fatty acids; SFAs, saturated fatty acids; MUFAs, mono unsaturated fatty acids; PUFAs, poly unsaturated fatty acids; FAs, fatty acids

*All intakes are rounded to the second decimal place

### Relationship between fatty acid intake and birth outcome

In a multiple regression analysis (Table 3)—after adjusting for maternal age, prepregnancy BMI, education level, neonatal gender, log-transformed maternal urinary cotinine level, gestational age at delivery, energy intake, and vitamin C intake (only for birth length)—total maternal omega 6 FA intake was negatively associated with birth weight (*β* = − 0.161; *P* = 0.031) and length (*β* = − 0.033; *P* = 0.025).

**Table 3:** Coefficients from multiple regression analysis between maternal fatty acids intakes with birth weight and birth length

	Birth weight(*n* 1291)						Birth length(*n* 1201)					
	Unadjusted			Adjusted^a^			Unadjusted			Adjusted^b^		
	*β*	SE	*P* value	*β*	SE	*P* value	*β*	SE	*P* value	*β*	SE	*P* value
TFAs	−0.009	0.463	0.985	−0.243	0.575	0.673	0.000	0.003	0.981	−0.001	0.004	0.806
SFAs	0.452	1.180	0.702	0.336	1.359	0.805	0.003	0.007	0.711	0.003	0.009	0.719
MUFAs	−0.057	1.059	0.957	−0.478	1.252	0.703	0.001	0.006	0.903	−0.001	0.008	0.951
PUFAs	−1.196	1.774	0.501	−2.847	2.109	0.177	−0.010	0.011	0.340	−0.017	0.014	0.199
Total *n*-3 FAs	0.965	8.785	0.913	−0.075	8.679	0.993	−0.058	0.052	0.261	−0.061	0.053	0.249
18:3 *n*-3	−12.306	15.416	0.425	−17.617	15.971	0.270	−0.131	0.092	0.155	−0.152	0.100	0.129
20:5 *n*-3	43.365	34.607	0.210	31.952	33.022	0.334	0.016	0.206	0.938	0.064	0.206	0.754
22:6 *n*-3	14.954	14.550	0.304	10.818	13.442	0.421	0.017	0.091	0.850	0.005	0.089	0.957
Total *n*-6 FAs	−2.946	2.076	0.156	−5.161	2.394	0.031^*^	−0.017	0.012	0.158	−0.033	0.015	0.025^*^
18:2 *n*-6	−2.315	1.953	0.236	−4.246	2.266	0.061	−0.011	0.012	0.332	−0.018	0.015	0.209
20:4 *n*-6	2.887	125.055	0.982	−67.266	121.643	0.580	0.002	0.760	0.998	−0.433	0.782	0.580
*n*-6/*n*-3	0.117	2.489	0.963	−0.439	2.437	0.857	0.013	0.015	0.398	0.015	0.015	0.304

TFAs, Total fatty acids; SFAs, saturated fatty acids; MUFAs, mono unsaturated fatty acids; PUFAs, poly unsaturated fatty acids

*P < 0.05

^a^adjusted for maternal age, pre-pregnancy BMI, level of education, neonatal gender, maternal urinary cotinine level (ln), gestational age and energy intake

^b^adjusted for maternal age, pre-pregnancy BMI, level of education, neonatal gender, maternal urinary cotinine level (ln), gestational age, energy and vitamin C intake

As shown in Table 4, the multiple logistic regression analysis with covariates revealed that the odds ratio for a lower birth weight (below the 10th percentile) was significantly high in the 5th quintile compared with that in the 1st quintile of total omega-6 FA intake (OR = 2.444; 95% CI = 1.308–5.751; *P* for trend = 0.010). In addition, the odds ratio for a greater birth length (above the than 90th percentile) was significantly low in the 5th quintile compared with that in the 1st quintile of total omega-6 FA intake (OR = 0.432; 95% CI = 1.308–5.751; *P* for trend = 0.020). When we further analyzed the relationship between the %E of n-6 FA intakes and birth weight and length, no significant association was found in multiple regressions. However, when we based our analysis on the recommended level of n-6 FA intakes for Korean (4–10%E), the odds ratio (OR) for the risk of being below the 10th percentile for birth weight was higher in the > 10%E (Q5) than in the < 4%E for omega-6 FA intake levels (OR; 4.013 CI; 1.199–3.432) (data not shown).

**Table 4:** Odds ratio (OR) and 95% confidence interval (CI) of birth weight and birth length with quintile of omega 6 intakes in the subjects

		*n*-6 FA intakes, quintiles (range, g/d)					
		Q1	Q2	Q3	Q4	Q5	*P* for trend
		(0.44–5.70)	(5.70–8.07)	(8.07–10.76)	(10.76–15.16)	(15.16–47.57)	
Birth weight (*n* 1078)							
For below 10th% (*n* 111)							
Unadjusted	OR	1.00	0.797	0.717	0.728	1.554	0.424
	95% CI		0.420–1.510	0.371–1.383	0.377–1.405	0.885–2.730	
Adjusted^a^	OR	1.00	0.666	0.680	0.939	2.444	0.010^*^
	95% CI		0.302–1.468	0.293–1.574	0.418–2.108	1.038–5.751	
For above 90th% (*n* 116)							
Unadjusted	OR	1.00	1.026	0.834	0.725	0.611	0.078
	95% CI		0.583–1.807	0.462–1.507	0.393–1.338	0.326–1.146	
Adjusted^a^	OR	1.00	0.974	0.633	0.716	0.539	0.109
	95% CI		0.517–1.836	0.316–1.268	0.350–1.465	0.237–1.224	
Birth length (*n* 1008)							
For below 10th% (*n* 100)							
Unadjusted	OR	1.00	0.951	0.994	0.476	0.968	0.593
	95% CI		0.508–1.779	0.531–1.861	0.224–1.010	0.521–1.799	
Adjusted^b^	OR	1.00	0.694	1.163	0.501	1.300	0.597
	95% CI		0.320–1.503	0.546–2.476	0.200–1.250	0.534–3.165	
For above 90th% (*n* 184)							
Unadjusted	OR	1.00	0.917	0.963	0.921	0.509	0.015^*^
	95% CI		0.563–1.493	0.591–1.570	0.562–1.509	0.296–0.874	
Adjusted^b^	OR	1.00	0.985	0.902	0.910	0.432	0.020^*^
	95% CI		0.559–1.735	0.501–1.655	0.501–1.655	0.211–0.884	

*P < 0.05

^a^Adjusted for maternal age, pre-pregnancy BMI, level of education, neonatal gender, maternal urinary cotinine level (ln), gestational age and energy intake

^b^Adjusted for maternal age, pre-pregnancy BMI, level of education, neonatal gender, maternal urinary cotinine level (ln), gestational age, energy and vitamin C intake

## Discussion

In our study, fetal growth was inversely associated with maternal omega-6 FA intake, because, after confounding variables were corrected, birth weight decreased with increasing maternal omega-6 FAs intake during pregnancy. In addition, when the maternal omega-6 FA intake was divided into quintiles, the odds ratio for a birth weight below the 10th percentile was significantly higher in the 5th quintile (Q5), which represents a higher intake of omega-6 FAs than in the 1st quintile (OR = 2.444; 95% CI = 1.308–5.751; *P* for trend = 0.010). Maternal omega-3 FAs intake and omega-6/omega-3 ratio, however, were not associated with birth weight or length.

The results of our study, showing that maternal omega-6 FAs intake is inversely related to birth weight, are quite unlike those of other studies; therefore it was difficult to find similar results. However, a recent report from India states that maternal intake of LA, which accounts for a large proportion of omega-6 FAs, is inversely related to birth weight. In that study (although not statistically significant but similar to our study), the birth weight was low when the maternal LA intake was high. In addition, birth weight was lower in the low maternal LA intake group, indicating an inverted U–shaped relationship between maternal LA intake and birth weight [15]. In our study, the relationship between maternal LA intake and birth weight was not statistically significant but showed a weakly negative relationship (*P* = 0.061), similar to the study in India.

Western diets are typically high in omega-6 FAs and low in omega-3 FAs, while the average intake of omega-3 FAs among our subjects was significantly higher. Therefore, omega-6 FAs may have different effects on pregnancy outcomes. Previous studies have also reported that birth weight is affected by race [34, 35]. The Danish study reported that pregnant women who consumed a Western diet that contained high omega-6 FAs had a high SGA baby birth rate, suggesting that omega-6 FAs intake may also influence the birth weight among Western people [36]. However, several studies [6, 37, 38] that reported increased birth size due to supplementation of omega-3 FAs showed that omega-3 FAs intake in Western populations plays an important role in pregnancy outcomes. Maternal increased intake of omega-6 FAs at high levels of omega-3 FAs intake among our subjects resulted in low birth weight, and that involved different conditions from previous studies for Western women.

The mechanisms of the relationship between maternal intake of omega-6 FAs and infant birth weight is not well established. However, some investigators have indeed found that pregnant women who had higher levels of total omega-6 FAs and ARA in their blood at late pregnancy delivered low-birth-weight babies [18, 39]. In addition, others have observed an association between higher levels of omega-6 FAs and ARA with reduced birth weight in early pregnancy [1, 17]. The elongation and desaturation of omega-6 and omega-3 FAs involve the same enzyme [40], indicating that high levels of omega-6 FAs can inhibit the conversion of omega-3 FAs. The mechanism of omega-3 FAs in improving fetal growth may be related to its effect on the endothelium [41]. The omega-3 LCPUFAs improves membrane fluidity [42] and receptor activity by increasing flow-mediated vasodilation in young adults [43], so it is possible that these FAs may increase the fetal growth rate by increasing placental blood flow and decreasing blood viscosity through the production of omega-3 FAs metabolite, such as prostacyclin and thromboxane [44]. This suggests that the decrease in birth weight when omega-6 FAs intake was high may be related to the inhibition of the positive effects of omega-3 FAs on fetal growth. Among our subjects, n-3 FAs intake and the omega-6/omega-3 ratio in fifth quintile were 2.86 ± 1.75 g and 9.12 ± 3.41, respectively, which were within FAO/WHO recommendations. However, the subtype of omega-3 FAs intake was mainly alpha-linolenic acid (ALA) among our subjects (Table 2). Theoretically, ALA, which is the essential fatty acid, is synthesized to DHA in the liver [45], but since ALA is hardly converted to DHA in young women [46, 47], it is doubtful whether the adequate DHA supply for fetal development was efficient. An intervention study [48] reported that the addition of ALA in the maternal diet did not increase the DHA level for the baby, suggesting that maternal direct intake of DHA would be more beneficial to the fetus. Our subjects had a high intake of omega-3 FAs but mostly ALA as mentioned, so it seemed difficult to show the positive effects of DHA or EPA in pregnancy outcomes. In addition, the average intake of omega-6 FAs in the fifth quintile was 21.35 ± 5.75 g, which was approximately 50% of the total fat intake, suggesting that excessive omega-6 FAs intake may be related to the inhibition of fatty acids synthesis such as DHA and EPA, consequently suppressing the positive effects of DHA in fetal growth.

Our study did not show any association between maternal intake of omega-3 FAs and birth weight. Some studies relating maternal intake of omega-3 FAs during pregnancy with birth outcome report a positive association between maternal omega-3 FAs intake and birth size [14, 49–51], while others have found no association [12, 52] or a negative association [53]. The study of pregnant women in India reported that birth weight was greater with a high intake of omega-3 FAs [15], suggesting the importance of omega-3 FAs intake for pregnant women in India. Our results, which showed no relationship between the maternal intake of omega-3 FAs and fetal growth, are probably explained by the relatively high intake of omega-3 FAs (mean intake 1470 mg per day, range from 40 to 11,880 mg) among our subjects. By comparison, the intake of omega-3 FAs by pregnant women was 11.49 mg per day in India [54] and 147 mg per day in the United Kingdom [12], both significantly lower than those in our study. Therefore it is important to determine whether the extremely low omega-3 FA intake among pregnant women in Korea is related to negative pregnancy outcomes.

The average fat intake of our subjects was 43.5 g (23.6% of the energy intake, EI), which is lower than the 71.8 g (31.9% of EI) in the United States [55], 99.1 g (29% of EI) in Mexico [56], and 74 g (31% of EI) in Denmark [57]; otherwise the fat intake in India (24% of EI) was similar [15]. Also, our subjects consumed 5.2% and 0.8% of energy in the form of omega-6 and omega-3 FAs respectively, which is within the range for omega-6 and omega-3 FAs recommended for pregnant women by the Food and Agriculture Organization/World Health Organization [58]. Our subjects in Q5 (comprising those with the highest intake of the omega-6 FAs), incidentally, consumed more than 10% (range from 7.7% to 24.3%) of their energy as omega-6 FAs. The mean omega-6/omega-3 ratio of our subjects was 9.8; it was 27.5 among pregnant women in India [15] and 12.3 among those in Mexico [56], indicating the omega-6/omega-3 ratios in our study were lower than those in other countries. However, this is explained by the fact that the omega-3 FAs intake of pregnant women in other countries is very low, while that of pregnant women in Korea is high. Nevertheless the omega-6/omega-3 ratio of our subjects was somewhat high when compared with the recommended standards [59]; therefore we concluded that it would be important to improve the dietary patterns of pregnant women in Korea.

This study has several limitations. First, it was difficult to accurately reflect the daily intake because the dietary intake of pregnant women was measured by a 1-day 24-h recall survey. However, in order to improve the accuracy of this method, we conducted a dietary survey employing trained dietitians using standard protocols, which helped the subjects reflect on their daily diets in detail, and we asked subjects whether their meal on the day of the dietary survey was the same as usual to reduce within-person variations. In addition, a study that was part of the Korean Health and Nutrition Survey showed that the values of fat intake obtained in each interview did not differ much from those of an additional one-day, 24-h recall and the original one-day diet interview, which was 2.5% [60]. Second, the results of FAs supplementation in our study were not available owing to the lack of data on omega-3 FAs– and omega-6 FAs–related products that might be consumed by our subjects. In our study, more than 50% of our subjects were taking supplements, and 57% of the supplements were folate supplements and iron supplements. Also, 36% of the supplements included prenatal multi supplements. Among the supplement users, only 3% of the pregnant women were consuming supplements containing omega FAs. Considering the fact that supplementation of omega-3 FAs such as DHA can affect pregnancy outcomes, further studies are needed to investigate the intake of omega-3 FAs through supplements. Finally, we lacked the data on omega-3 FAs and omega-6 FAs in the plasma, which could have substantiated the relationship observed between maternal omega-6 FAs nutrition and birth weight due to the limited availability of samples for other measurements (environmental heavy metals, toxins, etc.). Measuring the plasma PUFAs levels and analyzing their relationship with pregnancy outcomes is important to further support the results of our study. Therefore, we will consider measuring plasma FAs for the future studies. Nevertheless these limitations, this study is the first, comprising a large cohort, to examine FAs intake among pregnant women in Korea and the relationship of FAs intake with pregnancy outcome. Based on this study, future studies are needed to elucidate how an excessive intake of omega-6 FAs is related to pregnancy outcome. It will also be important to suggest ways in which to limit the consumption of omega-6 FAs among pregnant women in Korea.

## Conclusion

This study was the first to investigate the relationship between the intake of FAs and pregnancy outcome among pregnant women in Korea. Despite adequate levels of omega-3 FAs, women who had high levels of omega-6 FAs tended to have lower birth weight infants. Therefore, reducing excessive omega-6 FAs intake of pregnant women in Korea will improve maternal nutritional status and also have more positive outcomes of pregnancy.

## Abbreviations

ARA: Arachidonic acid
DHA: docosahexaenoic acid
FAs: Fatty acids
LA: Linoleic acid
LC-PUFAs: Long-chain polyunsaturated fatty acids
MOCEH: Mothers’ and Children’s Environmental Health
MUFAs: Mono unsaturated fatty acids
n-6/n-3: Ratio of n-6-to-n-3 polyunsaturated fatty acids
PUFAs: Polyunsaturated fatty acids
SFAs: Saturated fatty acids
TFAs: Total fatty acids
